# Neuroendocrine and metabolic components of dopamine agonist amelioration of metabolic syndrome in SHR rats

**DOI:** 10.1186/1758-5996-6-104

**Published:** 2014-09-25

**Authors:** Michael Ezrokhi, Shuqin Luo, Yelena Trubitsyna, Anthony H Cincotta

**Affiliations:** VeroScience LLC, Tiverton, RI 02878 USA

**Keywords:** Neuroendocrine, Bromocriptine, Diabetes, Insulin resistance, Resetting

## Abstract

**Background:**

The hypertensive, pro-inflammatory, obese state is strongly coupled to peripheral and hepatic insulin resistance (in composite termed metabolic syndrome [MS]). Hepatic pro-inflammatory pathways have been demonstrated to initiate or exacerbate hepatic insulin resistance and contribute to fatty liver, a correlate of MS. Previous studies in seasonally obese animals have implicated an important role for circadian phase-dependent increases in hypothalamic dopaminergic tone in the maintenance of the lean, insulin sensitive condition. However, mechanisms driving this dopaminergic effect have not been fully delineated and the impact of such dopaminergic function upon the above mentioned parameters of MS, particularly upon key intra-hepatic regulators of liver inflammation and lipid and glucose metabolism have never been investigated.

**Objective:**

This study therefore investigated the effects of timed daily administration of bromocriptine, a potent dopamine D2 receptor agonist, on a) ventromedial hypothalamic catecholamine activity, b) MS and c) hepatic protein levels of key regulators of liver inflammation and glucose and lipid metabolism in a non-seasonal model of MS - the hypertensive, obese SHR rat.

**Methods:**

Sixteen week old SHR rats maintained on 14 hour daily photoperiods were treated daily for 16 days with bromocriptine (10 mg/kg, i.p.) or vehicle at 1 hour before light offset and, subsequent to blood pressure recordings on day 14, were then utilized for in vivo microdialysis of ventromedial hypothalamic catecholamine activity or sacrificed for the analyses of MS factors and regulators of hepatic metabolism. Normal Wistar rats served as wild-type controls for hypothalamic activity, body fat levels, and insulin sensitivity.

**Results:**

Bromocriptine treatment significantly reduced ventromedial hypothalamic norepinephrine and serotonin levels to the normal range and systolic and diastolic blood pressures, retroperitoneal body fat level, plasma insulin and glucose levels and HOMA-IR relative to vehicle treated SHR controls. Such treatment also reduced plasma levels of C-reactive protein, leptin, and norepinephrine and increased that of plasma adiponectin significantly relative to SHR controls. Finally, bromocriptine treatment significantly reduced hepatic levels of several pro-inflammatory pathway proteins and of the master transcriptional activators of lipogenesis, gluconeogenesis, and free fatty acid oxidation versus control SHR rats.

**Conclusion:**

These findings indicate that in SHR rats, timed daily dopamine agonist treatment improves hypothalamic and neuroendocrine pathologies associated with MS and such neuroendocrine events are coupled to a transformation of liver metabolism potentiating a reduction of elevated lipogenic and gluconeogenic capacity. This liver effect may be driven in part by concurrent reductions in hyperinsulinemia and sympathetic tone as well as by reductions in intra-hepatic inflammation.

**Electronic supplementary material:**

The online version of this article (doi:10.1186/1758-5996-6-104) contains supplementary material, which is available to authorized users.

## Introduction

Many vertebrate species in the wild exhibit annual cycles of metabolism, oscillating between seasons of obese, insulin resistance and lean, insulin sensitivity (reviewed in [1, 2]. The ability to anticipate a season of low food availability by the endogenous induction of the obese, insulin resistant state supports survival during such a subsequent season when food availability is scarce. During the obese, insulin resistant season, in the absence of ample carbohydrate in the environment, increased hepatic glucose output supports brain function while fat stores are utilized in insulin resistant peripheral tissues thereby sparing plasma glucose for utilization by the brain, which has a near absolute requirement for glucose as a fuel. The circadian rhythm of dopamine release at the region of the biological clock, the hypothalamic suprachiasmatic nuclei (SCN), has been implicated in the regulation of peripheral insulin sensitivity and glucose and lipid metabolism in such seasonal mammals (reviewed in [1, 2]). The circadian peak of dopamine release at the region of the SCN in seasonal insulin sensitive animals is absent in seasonal insulin resistant animals [3] and ablation of this dopaminergic activity by SCN area site-specific neurotoxin application in seasonal or nonseasonal insulin sensitive animals induces a marked insulin resistant state [4]. Moreover, intraperitoneal or intracerebroventricular administration of bromocriptine, a potent dopamine D2 receptor agonist, to seasonal insulin resistant animals reverses the insulin resistance/glucose intolerance [5–7]. Such bromocriptine treatment has been demonstrated to reduce both hepatic glucose and lipid production and secretion in seasonal insulin resistant animals [6, 8–10]. Other studies have identified the ventromedial hypothalamus as an additional target for such metabolic influences of hypothalamic dopaminergic activity (potentially in concert with or resulting from such activity at the region of the SCN). Bromocriptine treatment of seasonal animals reduces elevated ventromedial hypothalamus (VMH) norepinephrine (NE) and serotonin (S) neuronal activities [2] that are characteristic of the insulin resistant state across a variety of animal models of the condition [1] and that can induce the obese, insulin resistant condition in normal animals (via VMH NE and S infusion) through their regulation of the neuroendocrine axis (e.g., simultaneous increase in sympathetic tone, plasma insulin, glucagon and norepinephrine among other factors) without alteration of the diet [1, 11, 12].

However, we are still searching for insights into how these (hypothalamic or other) influences of bromocriptine affect regulatory biochemical pathways in liver that facilitate/mediate these simultaneous bromocriptine effects on glucose and lipid metabolism therein and systemically. Also, it is not known whether such bromocriptine effects on VMH neurochemistry are specific to seasonal animals or are a fundamental-general phenomenon of the insulin resistant state among mammals, across seasonal and non-seasonal animal models of insulin resistance alike. Moreover, the potential impact of bromocriptine treatment not merely on insulin resistance but rather upon the MS (insulin resistance, obesity, dyslipidemia, hypertension, and hepatic inflammation) has not been fully investigated. This study therefore investigated the impact of bromocriptine treatment in the hypertensive, insulin resistant SHR rat on a) VMH neurochemistry known to regulate peripheral (hepatic) glucose and lipid metabolism, b) the broader malaise of MS (hypertension, obesity, fatty liver, hyperinsulinemia, insulin resistance, and pro-inflammatory state) that contribute to cardiometabolic risk and c) liver biochemical pathways operative in the (dys)regulation of hepatic glucose and lipid production, namely transcription factor or enzyme proteins modulating proinflammatory (SOCS3, NFκB, IKK, JNK), gluconeogenic (FOXO1-Ser256, PEPCK, G6Pase, PGC-1α) fatty acid oxidative (PGC-1α, PPARα), and lipogenic (SREBP-1, mTORC, PGC-1β, PPARγ) activities. Here we show that dopamine agonist treatment with bromocriptine at the onset of locomotor activity in SHR rats normalizes elevated levels of VMH noradrenergic and serotonergic activities associated with and known to induce insulin resistance and fattening in seasonal rodents. Such treatment also reduces hypertension, insulin resistance, fatty liver and several key hepatic transcription factors that induce hepatic pro-inflammatory pathways, gluconeogenesis and lipogenesis. The bromocriptine-induced reduction in hypertension is associated with and may be potentiated by its induced changes in VMH activity and reduced sympathetic tone. The bromocriptine-induced reduction in liver and adipose fat content is associated with and may derive in part from reductions of hyperinsulinemia, liver lipogenic responsiveness to insulin and pro-inflammatory pathways that potentiate lipogenic activity. The reduction in bromocriptine-induced insulin resistance is coupled to and may derive in part from reductions in hepatic transcription factors potentiating gluconeogenesis and fatty acid oxidation.

## Materials and methods

### Animals

Male spontaneously hypertensive rats (SHR) and Wistar rats (Taconic, Hudson, NY) were housed individually and habituated to our climate-controlled animal care facility (14 hour daily photoperiods, light onset at 05:00) for at least 14 days before initiation of any experimentation. They were allowed to feed (Standard lab chow 18.6% protein, 44.2% carbohydrate, and 6.2% fat rodent diet 2018, Harlan, NY) and drink ad libitum throughout the study period. At the initiation of experimentation, animals were 16 weeks of age and at an average body weight of 334 ± 4 grams. Male SHR rats of this strain and age are severely hypertensive [13].

### Experimental design

Two separate investigations were conducted in this study. In the initial investigation, we tested for possible differences between hypertensive SHR versus normal Wistar rats in extracellular monoamine (dopamine, serotonin, and norepinephrine) metabolites (measure of neurotransmitter release level) in the VMH that are known to be strongly involved in the regulation of insulin sensitivity, liver glucose output, plasma lipid level and body fat store level in rodents [1, 11, 12, 14]. This investigation also examined the potential impact of chronic bromocriptine administration upon these VMH monoamine profiles in SHR rats. In vivo microdialysis was employed to study daily extracellular profiles of monoamine metabolites in the VMH of SHRs treated with bromocriptine (10 mg/kg/day) (N = 8) or vehicle (N = 8) and vehicle treated, age-matched normotensive Wistar rats (N = 6) (administered at 13 hours after light onset [HALO]) for 14 days. Microdialysis samples from the VMH of free living rats held under a daily photoperiod and allowed to feed and drink ad libitum during the sampling were collected every 2 hours continuously over a 24 hour period. Microdialysis samples were assayed via HPLC for the metabolites of dopamine (homovanillic acid, HVA), serotonin (5-hydroxy-indoleacetic acid, 5HIAA), and norepinephrine (3-methoxy-4-hydroxy-phenylglycol, MHPG) as previously described [14].

In the second investigation, SHR rats were randomized to one of two treatment groups: bromocriptine (10 mg/kg/day) (N = 8) or vehicle (30% ethanol, USP) (N = 8) administered intraperitoneally at 13 HALO (a time of peak dopamine release at the area of the SCN clock in nocturnal rodents [4]) daily for 16 days. A group of Wistar rats served as the wild-type control for lipid, insulin resistance, and blood pressure measures. Measurements of blood pressure were taken at 4 hours after light onset on the day before treatment initiation and again 14 days after treatment (15 hours after the final bromocriptine injection). Animals were sacrificed on day 16 of the study at 4 HALO, the peak time of daily fasting in these nocturnal animals, and blood samples were collected for analyses of humoral metabolic and immune factors, including plasma insulin, glucose, leptin, adiponectin, norepinephrine, and C-reactive protein (CRP). Retroperitoneal fat pads were removed and weighed as an index of body fat store level and livers were quickly removed and frozen for quantitative analyses of proteins regulating inflammation ([nuclear factor kappa-light-chain-enhancer of activated B cells; NfκB], [IκB kinase; IKKαβ], [c-Jun N-terminal kinase; JNK], [suppressor of cytokine signaling; SOCS3]), gluconeogenesis ([peroxisome proliferator-activated receptor gamma coactivator-1α; PGC1α], [Forkhead box protein O1; FOXO1], [Glucose 6-phosphatase; G6Pase], [Phosphoenolpyruvate carboxykinase; PEPCK]), lipogenesis ([peroxisome proliferator-activated receptor gamma coactivator-1 beta; PGC1β], [sterol regulatory element-binding protein 1; SREBP1], [mammalian target of rapamycin complex 1; mTORC1], [peroxisome proliferator-activated receptor; PPARγ]), and fatty acid oxidation (PGC1α and PPARα), and for Total liver lipid content. Daily food consumption and body weight changes were monitored periodically over the course of the study. All animal experiments were conducted according to the protocols approved by the Institutional Animal Care and Use Committee of VeroScience, LLC.

### Blood pressure (BP) measurements

Systolic and diastolic blood pressure was measured on conscious animals with a tail-cuff Volume Pressure Recording method (CODA-6 non-invasive blood pressure system, Kent Scientific Corp. Torrington, CT) following manufacturer’s instructions. Several days before experimental recordings, rats were acclimated to the restraining cage and the tail cuff to minimize or reduce any stress influence on the readings. BP measurements were performed before and after 14 days of treatment. BP values per animal were the result of an average of 6-8 measurements.

### Surgery

After a two week adaptation to the facility, each rat in the microdialysis study was anesthetized with a mixture of ketamine and xylazine (80:60 mg/kg body weight, i.p.) and placed on a stereotaxic apparatus (David Kopf). A 30-gauge stainless steel guide cannula (Carnegie Medicine, Stockholm, Sweden) was permanently implanted aimed at the top of the VMH [15] at coordinates: 2.8 mm posterior to bregma, 0.7 mm right lateral to the midsagittal suture, and 9.0 mm ventral to the surface of the dura with the incisor bar set 1 mm below the interaural line. The cannula was secured permanently to the skull with three stainless steel screws penetrating the skull and acrylic cement.

### Microdialysis

During the entire microdialysis procedure, each animal was placed in an acrylic bowl with free access to food and water and was maintained on a 14-h daily photoperiod (light onset 05:00 am). A 32-gauge dialysis probe with a 1-mm-long tip of semi-permeable membrane (20,000 molecular weight cutoff) was inserted into the guide cannula and the probe membrane was placed in the VMH at 9.0-10.0 mm with respect to the dura. Using a microinjection pump (Carnegie Medicine, CMA/100), filtered Ringer’s solution (147 mM NaCl, 3.4 mM CaCl_2_, 4.0 mM KCl, pH 6.0) was continuously perfused through the probe at a rate of 0.12 μl/min. The probe was connected to the microinjection pump by microbore Teflon tubing through a counterbalanced 2-channel liquid swivel arm (Bioanalytical Systems, West Lafayette, IN) attached to the rim of the bowl, thus permitting the animal to move freely without the tubing becoming tangled during the experimental period. Rats were put in the bowl and attached to the microdialysis device at 10:00 hours (5 HALO) on the test day following 16 days of treatment and sample collection subsequently began at 5 hours after light onset. Microdialysis samples were collected into 300 μl vials (containing 2 μl of 0.1 N perchloric acid solution) at 2-hour intervals continuously for 24 hours through an automated refrigerated fraction collector (modified CMA/170, CMA/Microdialysis, Acton, MA, USA). Bromocriptine or vehicle was given 1 hour before light offset daily for 14 days including on the day of microdialysis.

### Analysis of microdialysis samples

The dialysis samples were analyzed by high-performance liquid chromatography with electrochemical detection (HPLC-EC with radial-flow cell; Bioanalytical System BAS 200). A 5 μl dialysate sample was injected into the system by an autosampler (Bioanalytical System Sample sentinel). The column used was a 100 × 2 mm reverse phase Octadecyl-silica ODS with 3 μm particle packing (BAS Unijet LC Column). The mobile phase contained 0.1 M monochloroacetic acid, 1.0 mM sodium octyl sulfate, 0.7 mM EDTA, 10 mM NaCl, and 2% (v/v) acetonitrile, adjusted to pH 3.1. The mobile phase was filtered and degassed before use, and delivered at a flow rate of 400 μl/min. A glassy carbon detector electrode (UniJet™ Amperometric Detector; BAS) was used for detection with an electrode oxidation potential of 0.65 V. Detector output was recorded on a computer. Peak integration and quantitation were performed via computerized software using an external standard for calibration. Standards were prepared in 0.1 N perchloric acid. Each of 5-HIAA, MHPG and HVA contents in the dialysate were determined. Results are reported as pg amine metabolite per 5 μl of dialysate.

### Assay of blood samples and analysis of liver lipid content

Blood glucose concentrations were determined by a blood glucose monitor (OneTouch Ultra, LifeScan, Inc, Milpitas, California). Plasma insulin, leptin, adiponectin, norepinephrine, and C-reactive protein (CRP) were assayed by EIA using commercially available assay kits utilizing anti-rat serum and rat insulin, leptin adiponectin, and CRP as standards (ALPCO Diagnostics, Salem, NH). Liver tissue was homogenized in 5% NP-40, heated, centrifuged, and supernatant assayed for triglyceride content by Triglyceride Determination Kit (Sigma-Aldrich, St. Louis, MO).

### Western blot analyses

Liver proteins were quantified by western blot analysis as follows using BioRad, (Hercules, CA) Criterion and ChemiDoc systems following manufacturers’ instructions. PGC1α antibody was purchased from EMD Millipore, Billerica, MA, Cat# ST1202. All other antibodies purchased from Santa Cruz Biotechnology, Santa Cruz, CA were as follows: FOXO1Ser256 Cat# sc-101681; G6Pase-α Cat# sc-27198; PEPCK Cat# sc-32879; PPARα Cat# sc-9000; SREBP-1 Cat# sc-13551; mTORC Cat# sc-8319; PPARγ Cat# sc-7273; PGC1β Cat# sc-67286, NFκB p65 Cat# sc-8008; IKKαβ Cat# sc-7607; SOCS3 Cat# sc-9023; JNK Cat# sc-571; Actin Cat# sc-47778. Criterion gradient tris-glycine precast gels, secondary antibodies, PVDF blotting membranes, molecular weight markers, and Enhanced Chemiluminescence (ECL) reagents were also purchased from BioRad. Samples from 8 SHR Vehicle, 8 SHR Bromocriptine treated rats, and 6 Wistar wild type controls were loaded onto the same 26 well Criterion gel along with the molecular weight markers; band intensity was compared only within the samples loaded onto the same gel. A housekeeping protein (actin) was concurrently quantified on all gels, and the test protein amount was normalized to actin in the Western blot analysis. Protein bands were quantified with BioRad ImageLab 4.1 software.

## Results

Effect of SHR vs Wistar background and of Bromocriptine in SHR rats on Daily VMH Extracellular Monoamine Metabolite Profiles.

**Figure 1:**
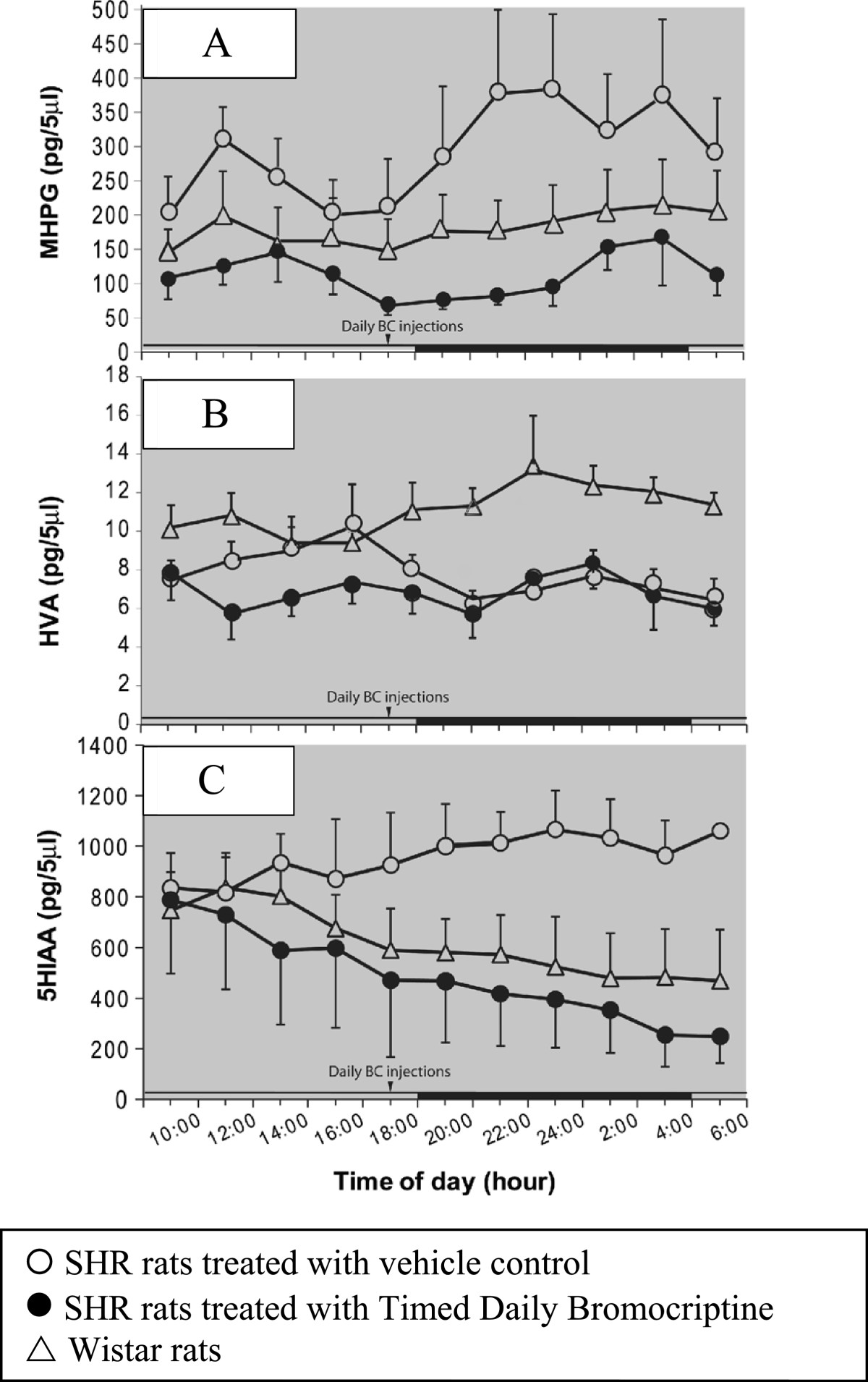
**Effect of timed daily bromocriptine administration on ventromedial hypothalamic catecholamines. A**. SHR rats exhibited elevated VMH NE release relative to Wistar normal controls (P < 0.0001). Timed daily bromocriptine administration significantly reduced VMH MHPG of SHR rats compared with vehicle treated SHR rats (P < 0.0001). **B**. SHR rats exhibited significantly lower extracellular VMH HVA content compared with normal Wistar rats (p < 0.001). **C**. SHR rats exhibited elevated VMH 5-HIAA release relative to Wistar normal controls (P < 0.0001). Timed daily bromocriptine administration significantly reduced VMH 5-HIAA of SHR rats compared with vehicle treated SHR rats (P < 0.0001) There was no significant difference in VMH 5-HIAA levels between SHR BC treated and Wistar rats.

Figure 1A shows the 22 hour pattern of extracellular MHPG in the VMH of freely moving SHR rats treated with either bromocriptine or vehicle for 14 days compared to that of normotensive Wistar controls. A two-way ANOVA with repeated measures revealed a significant main effect of the bromocriptine treatment (F_10, 80_ = 2.340, P < 0.05). SHR rats exhibited elevated VMH NE release relative to Wistar normal controls (66% increase, P < 0.0001). However, timed daily bromocriptine administration significantly reduced VMH MHPG of SHR rats compared with vehicle treated SHR rats (65%, P < 0.0001).

Figure 1B shows the 22 hour pattern of extracellular HVA in the VMH of freely moving SHR rats treated with either bromocriptine or vehicle for 14 days compared to that of normotensive Wistar controls. A two-way ANOVA with repeated measures revealed a significant strain treatment effect (F_2, 80_ = 10.069, P < 0.005) and a significant interaction effect between treatment and time of day (F_20, 80_ = 1.99, P < 0.02). SHR rats had significantly lower extracellular VMH HVA content compared with normal Wistar rats (29%, P < 0.001). However, extracellular VMH HVA content was not different between BC and vehicle treated SHR rats.

Figure 1C shows the 22 hour pattern of extracellular 5-HIAA in the VMH of freely living SHR rats treated with either bromocriptine or vehicle for 14 days compared to that of Wistar control animals. There was a significant effect of the interaction between treatment and time of day (F_20, 80_ = 2.555, P < 0.002) by a two-way ANOVA with repeated measures. Post-hoc multiple-range test revealed a significant group difference on VMH 5-HIAA levels between SHR vehicle treated and BC treated (49% reduction by BC treatment; P < 0.0001) as well as between SHR and Wistar vehicle treated rats (53% reduction in Wistars; P < 0.0001). There was however, no significant difference on VMH 5-HIAA levels between SHR BC treated and Wistar rats.

### Effects of bromocriptine on metabolic and neuroendocrine factors potentiating metabolic syndrome

**Figure 2:**
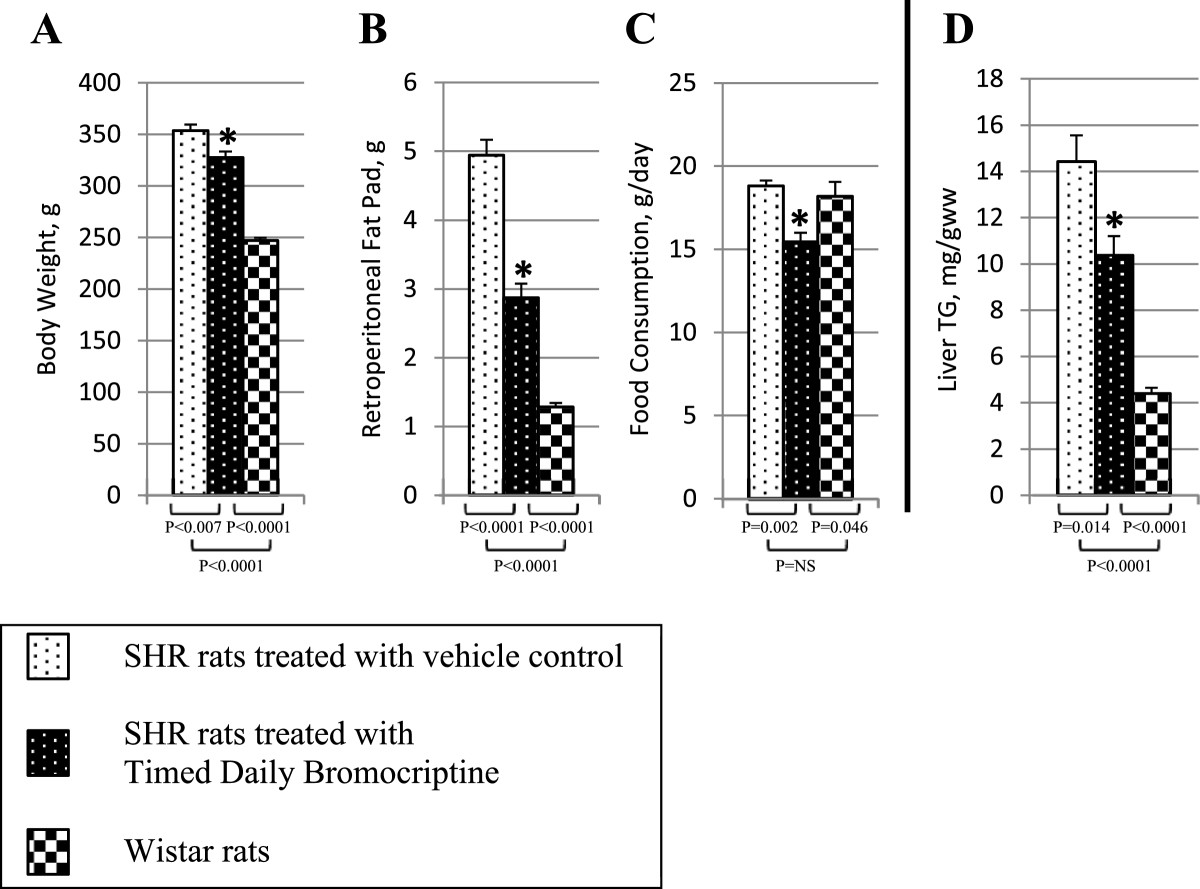
**Impact of timed daily bromocriptine or vehicle administration on body weight (Panel A), retroperitoneal fat pad (Panel B), food consumptions (Panel C) and liver triglycerides (Panel D).** Values are means ± SEM of 8 animals in each group. *Difference is statistically significant; P values are noted under each panel.

**Figure 3:**
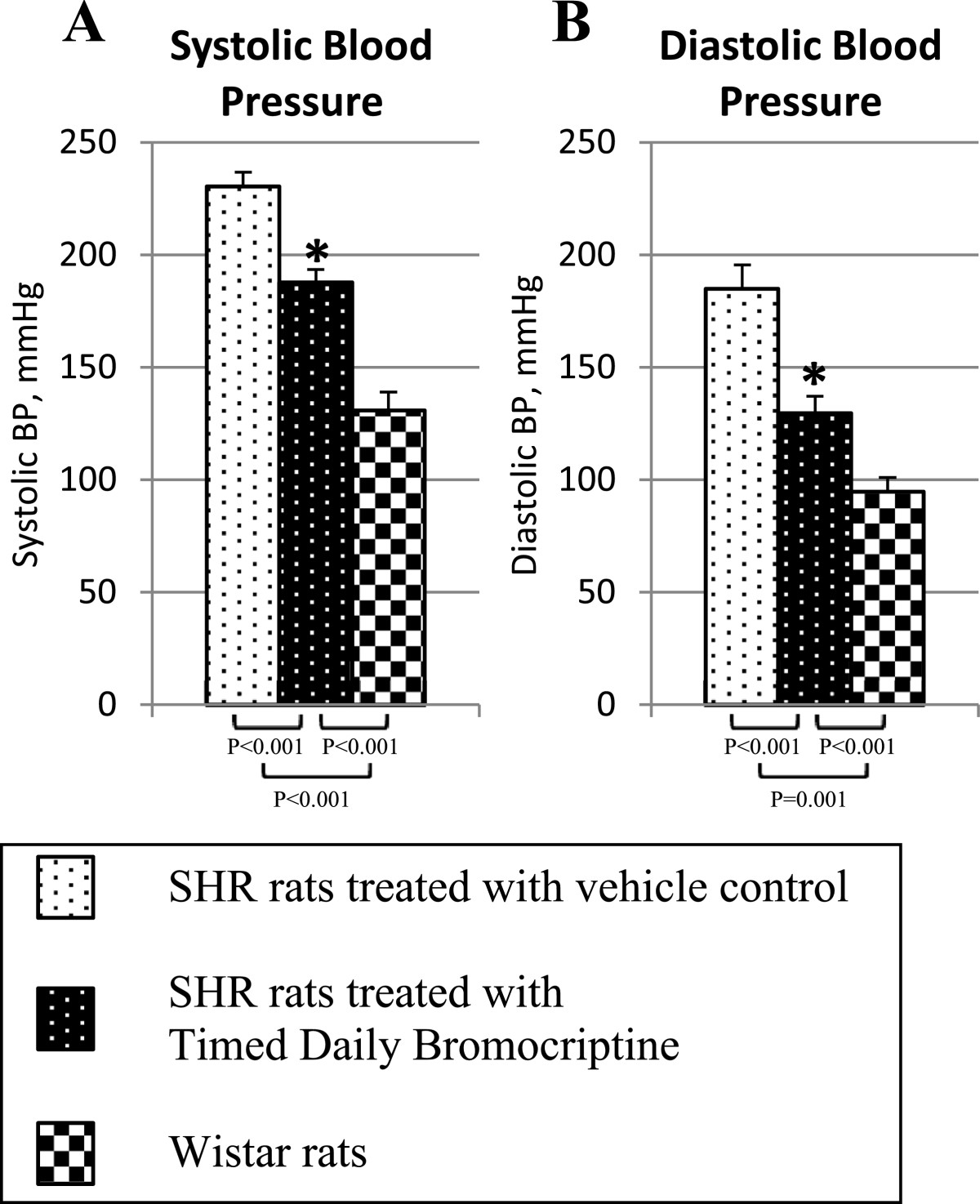
**Impact of timed daily bromocriptine or vehicle administration on systolic (Panel A) and diastolic (Panel B) blood pressure.** Values are means ± SEM of 8 animals in each group. *Difference is statistically significant; P values are noted under each panel.

**Figure 4:**
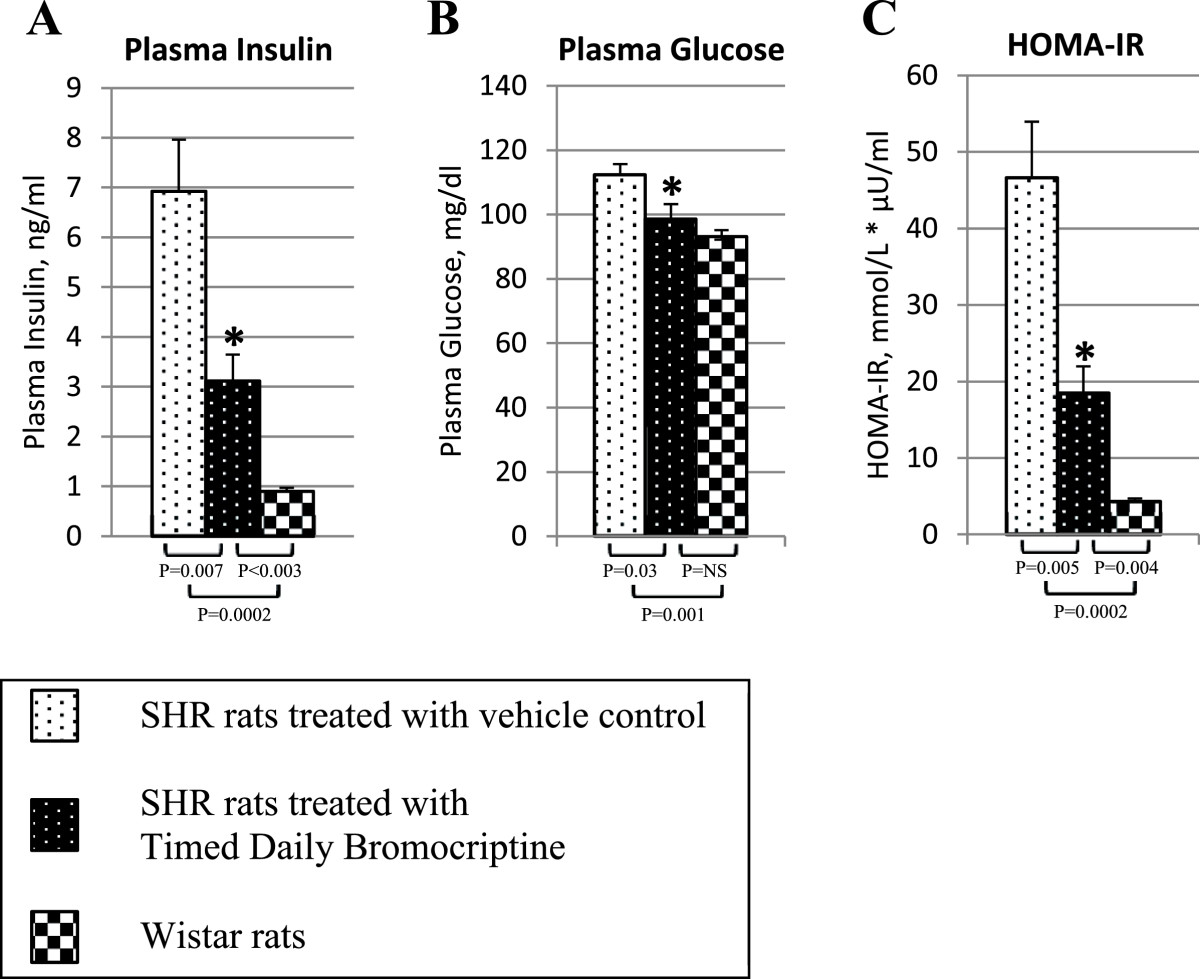
**Impact of timed daily bromocriptine or vehicle administration on plasma glucose (Panel A), plasma insulin (Panel B), and HOMA-IR index (Panel C).** Values are means ± SEM of 8 animals in each group. *Difference is statistically significant; P values are noted under each panel.

**Figure 5:**
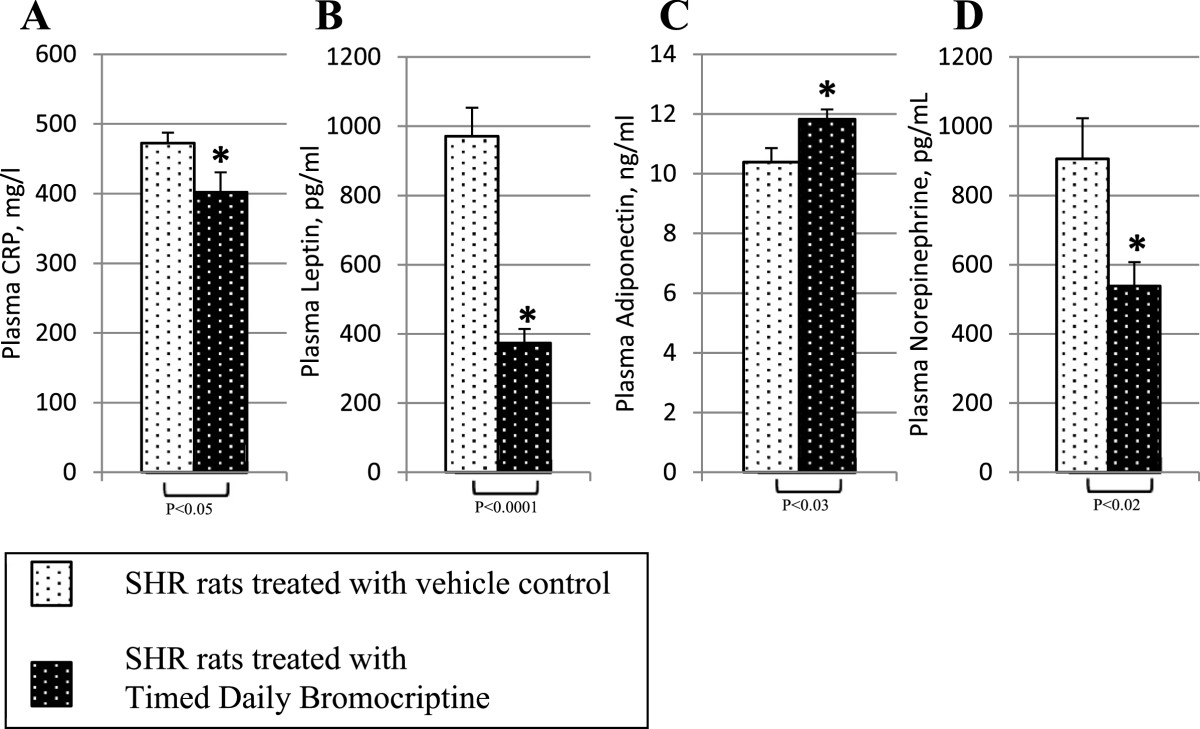
**Impact of timed daily bromocriptine or vehicle administration on humoral modulators of metabolism and inflammation – plasma CRP (Panel A), plasma leptin (Panel B), plasma adiponectin (Panel C), and plasma norepinephrine (Panel D).** Values are means ± SEM of 8 animals in each group. *Difference is statistically significant; P values are noted under each panel.

Timed daily bromocriptine treatment for 14 days significantly reduced retroperitoneal fat pad weight of SHR rats by 42% (P < 0.0001), body weight and average daily food consumption slightly over the 14 day treatment period relative to vehicle treated controls (Figure 2). Such bromocriptine treatment also significantly reduced liver lipid content by 28% (P = 0.01). Bromocriptine treatment also reduced systolic blood pressure from 230 ± 6 mmHg to 188 ± 6 mmHg (P = 0.0003) and diastolic blood pressure from 185 ± 11 mmHg to 130 ± 8 mm Hg (P = 0.001) towards the values observed in wild type Wistar rats (Figure 3).Bromocriptine treatment reduced plasma glucose concentrations by 12% (from 112 ± 3 to 99 ± 5 mg/dl, P = 0.03), and plasma insulin by 55% from 6.9 ± 1.0 to 3.1 ± 0.5 ng/ml (P = 0.007), resulting in a reduction in the homeostasis model assessment of insulin resistance (HOMA-IR) by 60% (from 47 to 18 μU/ml*mmol/L, P = 0.004) relative to vehicle controls towards the values observed in wild type Wistar rats (Figure 4). Bromocriptine treatment produced significant reductions in plasma leptin by 62% (from 971 ± 83 to 374 ± 41 pg/ml, P < 0.0001), plasma norepinephrine by 41% (from 906 ± 117 to 538 ± 69 pg/ml, P < 0.02), and CRP by 15% (from 472 ± 15 to 402 ± 29 mg/l, P < 0.05) levels, while it increased plasma adiponectin levels by 14% (from 10.4 ± 0.5 to 11.8 ± 0.3 ng/ml, P < 0.03) (Figure 5).

### Effect of bromocriptine on liver pro-inflammatory, gluconeogenic, fatty acid oxidative, and lipogenic proteins

**Figure 6:**
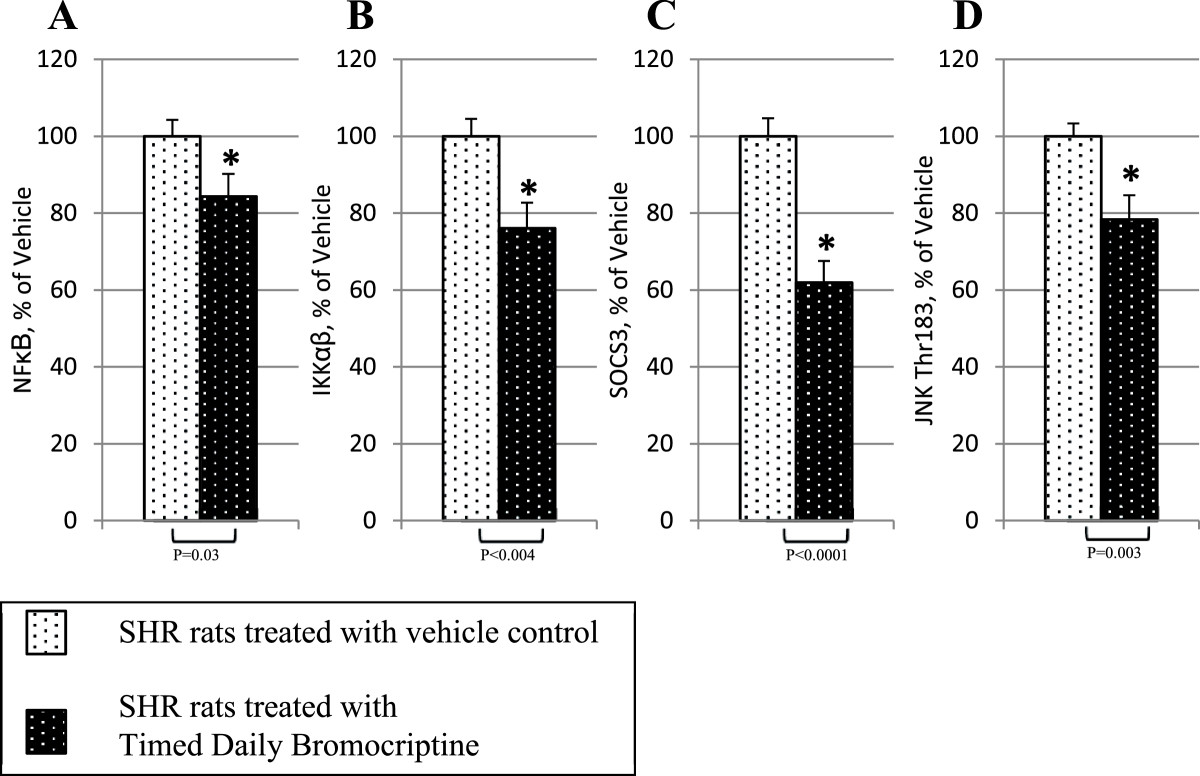
**Impact of timed daily bromocriptine or vehicle administration on pro-inflammatory regulators – NFκBp65 (Panel A), IKKαβ (Panel B), SOCS3 (Panel C), and JNK (Panel D).** Proteins we quantified by Western blotting. Values are means ± SEM of 8 animals in each group. *Difference is statistically significant; P values are noted under each panel.

**Figure 7:**
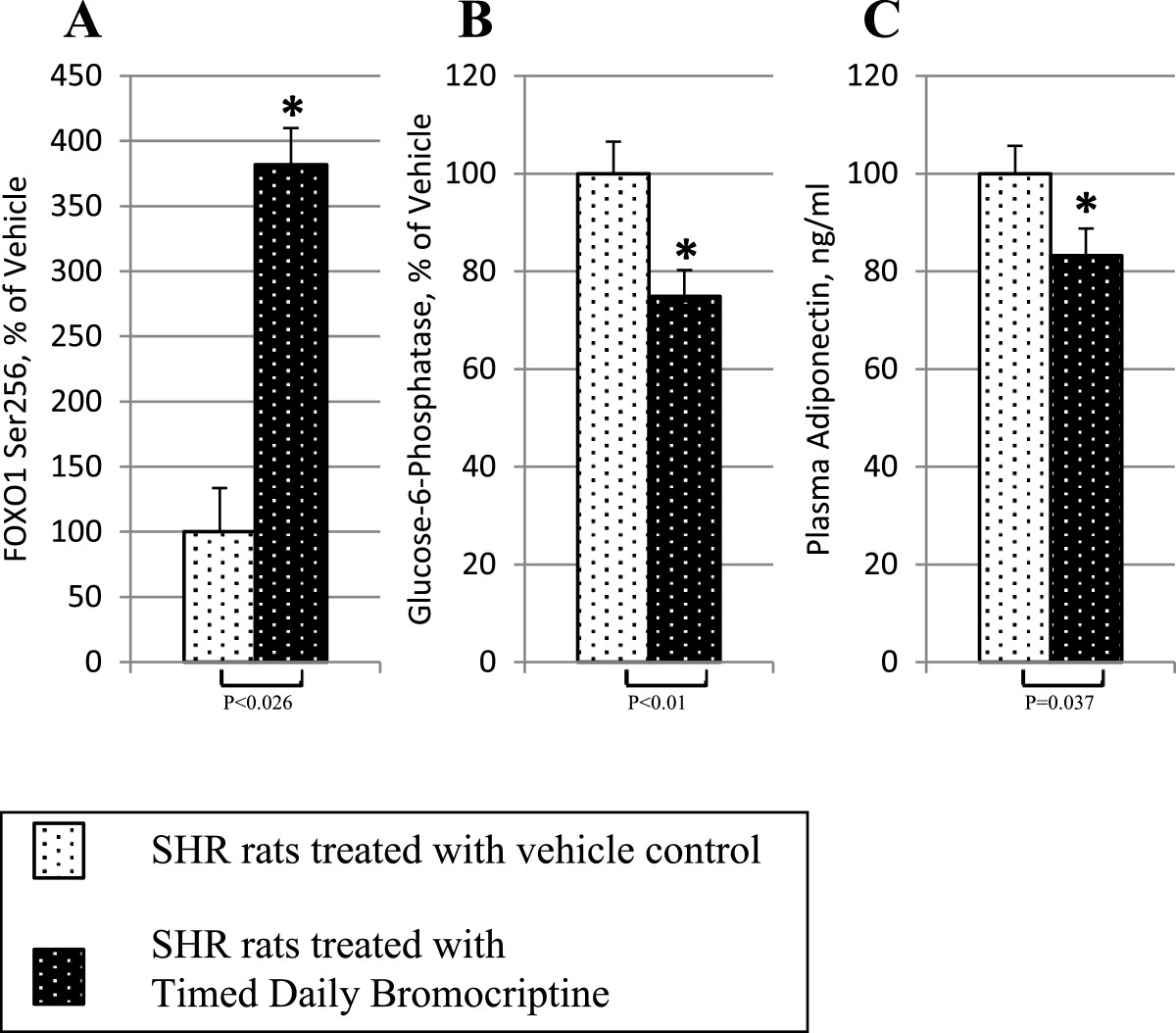
**Impact of timed daily bromocriptine or vehicle administration on gluconeogenic pathway regulators – FOXO1 phosphorylated at Ser256 (Panel A), glucose-6-phosphatase (Panel B), and PEPCK (Panel C).** Proteins we quantified by Western blotting. Values are means ± SEM of 8 animals in each group. *Difference is statistically significant; P values are noted under each panel.

**Figure 8:**
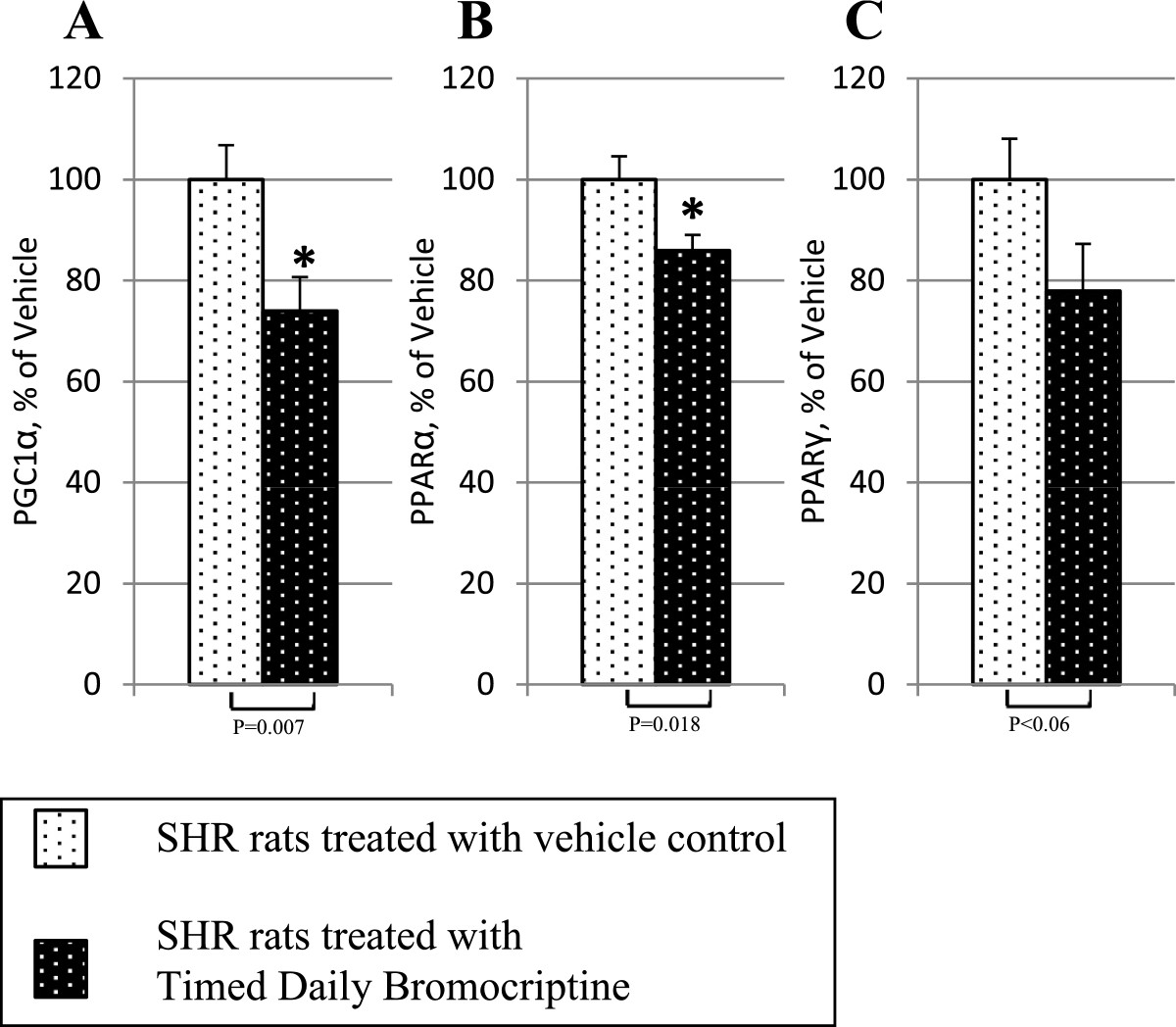
**Impact of timed daily bromocriptine or vehicle administration on fatty acid oxidation activators – PGC1α (Panel A), PPARα (Panel B), and PPARγ (Panel C).** Proteins we quantified by Western blotting. Values are means ± SEM of 8 animals in each group. *Difference is statistically significant; P values are noted under each panel.

**Figure 9:**
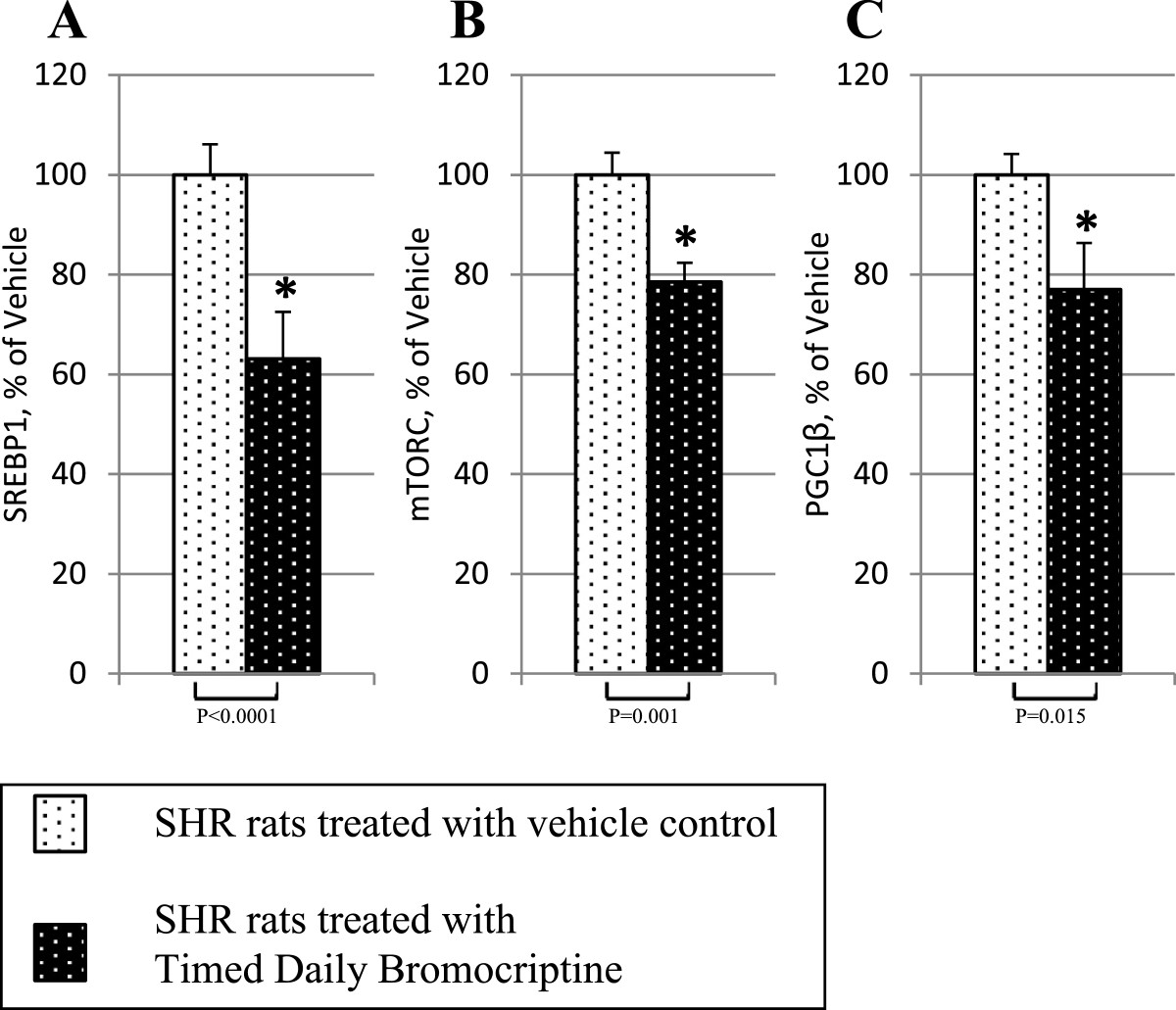
**Impact of timed daily bromocriptine or vehicle administration on lipogenesis regulators – SREBP1 (Panel A), mTORC (Panel B), and PGC1β (Panel C).** Proteins we quantified by Western blotting. Values are means ± SEM of 8 animals in each group. *Difference is statistically significant; P values are noted under each panel.

Bromocriptine treatment simultaneously reduced the levels of the liver inflammatory pathway activating transcription factor proteins NFκBp65, JNK, IKKαβ, and SOCS3 by 16% (P = 0.03), 22% (P = 0.003), 24% (P = 0.004), and 38% (P < 0.0001), respectively relative to control values (Figure 6). Such bromocriptine treatment also reduced the levels of the hepatic gluconeogenic enzymes, PEPCK and G6Pase while increasing the levels of inactivated ser256 phosphorylated FOXO1 (thereby reducing the stimulatory effect of FOXO1 on gluconeogenesis), by 17% (P < 0.04), 25% (P < 0.01), 282% (P = 0.026), respectively (Figure 7). Bromocriptine also reduced the hepatic levels of pro-FFA oxidative transcription factors, PGC1α, PPARα and PPARγ by 26% (P < 0.01), 14% (P < 0.02), and 22% (P = 0.06), respectively (Figure 8). Bromocriptine also reduced the levels of liver triglyceride synthesis promoting proteins SREBP1, mTORC1, and PGC1β by 37%, (P < 0.001), 21% (P = 0.001), and 23% (P = 0.015), respectively, relative to SHR control (Figure 9).

## Discussion

This study is the first to demonstrate that dopamine agonist treatment ameliorates both major central and peripheral components of MS as evidenced by a simultaneous improvement in a range of neuroendocrine and metabolic pathologies including hypertension, obesity, hyperglycemia, hyperinsulinemia, insulin resistance, hyperleptinemia, increased liver lipid content, and hepatic pro-inflammatory protein pathway activation. Available evidence suggests that such wide spread influences on metabolism are largely via the hypothalamic/neuroendocrine axis [1, 2, 10–12, 14]. Intracerebroventricular (icv) administration of bromocriptine to seasonal insulin resistant hamsters reduces body fat store levels, hyperinsulinemia, insulin resistance, and glucose intolerance. Information regarding the CNS targets for such icv dopamine agonist-induced improvements in seasonal insulin resistance indicates that modulation of monoamine activity at the hypothalamic VMH is at least one such CNS target [11, 12, 14] among others (reviewed in [1]). Seasonal insulin resistance is associated with elevations in VMH NE and 5HT activities and importantly, infusion of exogenous NE and 5HT to the VMH of seasonal insulin sensitive animals to raise these monoamine levels to those observed in seasonal insulin resistant animals induces marked insulin resistance and beta cell dysfunction [12, 15] and this event has also been observed in non-seasonal animals as well [11]. Systemic bromocriptine treatment normalizes these VMH monoamine dysfunctions and insulin resistance in seasonal hamsters [14]. Also, preliminary data indicate that this influence of bromocriptine treatment to reduce insulin resistance can be largely attenuated by concurrent infusion of NE into the VMH (Cincotta, unpublished data). The present observations of a) increased VMH NE and 5HT activities in hypertensive, insulin resistant SHR versus normal Wistar rats and b) concurrent reductions in these VMH monoamine activities and amelioration of MS extend and corroborate these previous findings now in a non-seasonal, genetic model of MS and suggest that such hypothalamic aberrations may be a fundamental component of the neuroendocrine milieu supporting induction of MS. Several other animal models of insulin resistance including the ob/ob mouse, db/db mouse, Zucker fatty rat, and offspring of malnourished or insulin treated pregnant rats all exhibit increased noradrenergic turnover within the VMH (reviewed in [1]).

Where and how bromocriptine is acting to produce these VMH corrections in monoamine activities is unknown, however sites involved may include at the VMH itself, as well as at the area of and surrounding the SCN [3, 4] the biological circadian pacemaker for the organism that communicates directly and indirectly (via the locus coeruleus and nucleus tractus solitarius) with the VMH and several other hypothalamic centers to regulate glucose tolerance [1]. Diminished dopaminergic activity at the area of the SCN is characteristic of the seasonal insulin resistant state that is coupled with elevated VMH NE and 5HT activity levels [3] and neurotoxic lesion of dopaminergic neurons within this SCN area of insulin sensitive animals induces seasonal insulin resistance [4]. Interestingly, the SHR rat is well characterized as having reduced dopaminergic tone within the prefrontal cortex and ventrolateral stiatum [16, 17] and the current study now indicates that such is also the case within the VMH itself. We are currently investigating whether dopaminergic activities at the SCN are also reduced in these SHR rats as in seasonal insulin resistant states. We have observed such reduced dopaminergic activity at the SCN subsequent to high fat diet-induced insulin resistance [18].

Elevated VMH NE and 5HT activity produces a unique neuroendocrine profile characterized by increased plasma NE, epinephrine, glucagon, insulin, free fatty acid (FFA), triglyceride, and leptin levels, increased sympathetic/neuroendocrine drive for hepatic glucose output, adipose lipolysis, and vasoconstriction [11], as well as an increased insulin secretory response to glucose and hyperinsulinemia [11, 15] that, as a composite, represents the hallmark of the MS. This neuroendocrine profile in turn is coupled to and/or potentiates insulin resistance, glucose intolerance, hyperlipidemia, obesity (reviewed in detail in [1] and hypertension [19]. Moreover, consistent with the observed normalization in elevated VMH NE and 5HT activities in this study, plasma levels of insulin, glucose, leptin, NE, and C-Reactive Protein were all reduced, and that of adiponectin was elevated following bromocriptine treatment in SHR rats. Furthermore, consistent as well with these neuroendocrine changes, HOMA-IR calculations indicate a marked improvement in insulin resistance following bromocriptine treatment. Additionally, plasma adiponectin levels are inversely correlated with and protect against both insulin resistance and fatty liver [20–22].

Likely due to decreased central (hypothalamic) dopaminergic tone as described above, SHR rats are hyperprolactinemic [23]. In turn, hyperprolactinemia has been demonstrated to potentiate fattening and insulin resistance in a variety of animal models and man (reviewed in [2]; [24–29]). In this regard it is critical to appreciate that the circadian rhythm of plasma prolactin level differs in lean, insulin sensitive versus obese, insulin resistant animals [2]. More importantly, a marked circadian rhythm of metabolic responsiveness to prolactin exits in vertebrates such that injections of prolactin into lean, insulin sensitive animals at the time of day its level peaks in the blood of obese, insulin resistant animals induces the obese, insulin resistant state while injections of prolactin into obese, insulin resistant animals at the time of day its level peaks in the blood of lean, insulin sensitive animals produces a lean, insulin sensitive condition (reviewed in [2], [30, 31]). Elevations of plasma prolactin levels can potentiate MS via decreasing hypothalamic dopamine release [32] thereby altering its signaling to the SCN to potentiate MS as described herein and elsewhere [1] that includes increasing lipogenic responsiveness to insulin in the periphery [33–35]. In the present study, bromocriptine, which is well known to effectively reduce hyperprolactinemia [36, 37], was administered daily just after the time of day of the normal circadian peak in plasma prolactin levels in lean, insulin sensitive rats [31] prior to the end of the photoperiod to re-establish the normal dopaminergic circadian input activity to the SCN. Such timed administration would also likely function to re-establish a more normal daily plasma prolactin profile as observed in insulin sensitive rats [31], though such plasma prolactin levels were not measured in this study. Consequently, the metabolic effects of timed daily bromocriptine treatment observed in this study may in part derive from a reduction of plasma hyperprolactinemia and normalization of the daily rhythm of plasma prolactin level towards that of lean, insulin sensitive rats.

It should be noted that several studies have defined an inhibitory effect of *acute* direct/autocrine/paracrine dopamine on beta cell glucose stimulated insulin secretion (GSIS) in animals and man [38–41], with a potential role for gastrointestinal L-DOPA as an endogenous source for such physiological beta cell dopamine responses that may potentiate hyperglycemia [41]. Moreover, acute administration of bromocriptine itself has been shown to produce such an inhibitory effect on beta cell GSIS potentially via noradrenergic α2 receptors [42]. While such observations of *acute* direct dopamine activity to inhibit beta cell GSIS and thereby potentiate hyperglycemia may seem at odds with a multitude of observations indicating that circadian timed *chronic* dopamine agonist therapy improves glucose intolerance and hyperglycemia, a careful consideration of the available evidence suggests a different scenario. In hyperinsulinemic glucose intolerant animals and man, chronic systemic timed bromocriptine treatment, that resets several hypothalamic circuits controlling metabolism [3, 4, 10–12, 14], reduces post glucose/meal challenge glucose area under curve (AUC) and insulin AUC simultaneously [5, 6, 10, 14, 43, 44]. Importantly, this effect in such animals can be manifested with intracerebroventricular administration of bromocriptine at nearly one thousandth the effective systemic dose [10], and likely involves bromocriptine’s effect to reduce elevated VMH NE and S activities and Paraventricular nucleus (PVN) Neuropeptide Y (NPY) and Corticotropin-releasing hormone (CRH) levels (present study, [11, 12, 14, 45], that potentiate insulin resistance, direct hyperinsulinemia independent of insulin resistance, and increased beta cell GSIS concurrently [1, 11, 12, 15]. However, in animals where glucose dysmetabolism progresses from impaired glucose tolerance to diabetes with beta cell dysfunction, such chronic dopamine agonist treatment reduces hyperglycemia while actually improving (increasing) GSIS, including the beta cell response to glucagon like peptide-1 [46, 47].

A plausible biological organization worth investigating that may unify these findings is the possibility that central (hypothalamic) circadian dopamine activities regulate beta cell function and insulin sensitivity in a coordinated fashion that would include regulation of paracrine dopamine activity at the beta cell. Under this postulate, in insulin resistant states, low hypothalamic dopamine activity allows for (potentiates) 1) an altered beta cell response to external (e.g., autonomic) stimuli that facilitates hyperinsulinemia and increased GSIS, as previous studies indicate [15], that may include low paracrine beta cell dopamine activity and 2) neuroendocrine mechanisms (as described in this study) facilitating insulin resistance to thereby establish a coordinated and controlled steady state hyperinsulinemic, insulin resistant condition (as observed in seasonal animals in the wild). Contrariwise, with appropriate circadian time increased hypothalamic dopaminergic activity, the hypothalamic metabolic control output coordinates an appropriate level of beta cell GSIS that may include increased (normal) paracrine dopamine activity and peripheral insulin sensitivity to maintain normal fasting and glucose tolerance glucose levels. Such a hypothalamic control system may contribute to the hyperbolic relationship of the “disposition index” that relates level of beta cell GSIS to insulin sensitivity [48] and we are currently investigating this possibility.

Thus, these bromocriptine-induced changes in the hypothalamic-neuroendocrine axis as a composite may in part contribute to the observed bromocriptine-induced decrease in hypertension, liver fat content and insulin resistance in these SHR rats as described below.

To appreciate the impact of bromocriptine treatment on hepatic lipid and glucose metabolism observed in this study, one must first review the physiological relationship between these liver activities in normal and insulin resistant states. The physiological relationship between hepatic lipid and glucose metabolism differs markedly between insulin sensitive and insulin resistant states (reviewed in [49]). Under normal physiological conditions, circadian increases in hepatic lipogenesis are generally coupled to decreases in whole body and hepatic FFA oxidation and hepatic glucose output typically synchronized to the fed state or feeding phase of the day. Contrariwise, circadian decreases in lipogenesis are coupled to increased whole body and hepatic FFA oxidation and hepatic glucose output during the fasted state or circadian fasting phase of the day in rodents [2]. Such circadian organization of these metabolic activities synchronizes the animal with its daily cyclic environment of food availability and internal locomotor activity rhythm (food seeking/gathering, feeding/lipogenesis synchronized temporally as are sleeping/fasting/FFA oxidation), thereby enhancing survival potential [2]. By comparison, in insulin resistant states, hepatic lipogenesis, FFA oxidation, and hepatic glucose output are all simultaneously increased at their respective circadian peak activity times (reviewed in [1]). In studies of humans as well, hepatic insulin resistance respecting control of glucose production associates most often with increased, not decreased, hepatic lipid synthesis/content and FFA oxidation [50–57]. Although a variety of genetic manipulations that result in increased hepatic FFA oxidation have been demonstrated to improve hepatic insulin resistance, primarily by decreasing hepatic lipid content (e.g., triacylglycerol, diacylglycerol, acyl-CoA, lysophosphatidic acid, and/or phosphatidic acid) [58–64], hepatic FFA oxidation is a potent stimulus for gluconeogenesis, reactive oxygen species generation (ROS), inflammatory cytokine production and insulin resistance [17, 19–22, 49–55, 57, 65, 66]. Moreover, reduction of hepatic FFA oxidation by genetic manipulations that actually enhance fatty liver is still coupled to improved insulin sensitivity or glucose tolerance [67–73], reviewed in [49]. In the SHR rat model investigated herein, as in human insulin resistant states such as obesity and fatty liver [50–55, 57], insulin resistance is coupled to increased hepatic lipid content, FFA oxidative activity/capacity, and gluconeogenic activity/capacity.

In obese, hyperinsulinemic, insulin resistant states such as the aged SHR rat, increased hepatic fatty acid level resulting from increased adipose lipolysis and subsequent plasma FFA uptake, dietary fat intake and/or de novo lipogenesis can potentiate increased inappropriate hepatic fat accumulation (e.g., steatosis) and fatty acid oxidation with ensuing cellular pathological consequences, termed lipotoxicity [49, 56, 65, 74–77], reviewed in [78, 79]. Lipotoxicity manifested as excess production of triacylglycerol intermediates (e.g., diacylglycerol, acyl-CoA, lysophosphatidic acid, phosphatidic acid) at specific and as yet poorly understood intracellular sites and/or overload in substrate driven fatty acid oxidation that ultimately results in incomplete fatty acid oxidation with the generation of oxidation intermediates (acid soluble intermediates) can facilitate reactive oxygen species (ROS) production and subsequent pro-inflammatory protein synthesis such as NFκB, JNK, and SOCS3 [65, 77, 80–82]. Such increased fatty acid oxidation also induces an increase in mitochondrial tricarboxylic acid cycle activity (though inadequate for the supply of fatty acids), increased gluconeogenic precursor generation, and subsequent respiratory dysfunction leading to increased generation of ROS [4, 8–10, 50–55, 57] that also stimulate such pro-inflammatory protein synthesis [55–57, 83–86] and stimulate PGC1 and FOXO1 synthesis [87], transcription factors that potentiate a further increased drive for FFA oxidation and gluconeogenesis [70, 74, 87–97]. Increases in hepatocellular levels of ROS and these pro-inflammatory proteins can also potentiate insulin resistance respecting insulin inhibition of gluconeogenesis and can simultaneously facilitate hepatic lipogenesis as more fully discussed below.

Regarding the influence of hepatic NFκB, JNK, and SOCS3 on gluconeogenesis, increased activity of any of these proteins can induce increased gluconeogenesis by inhibiting hepatic insulin signaling [53, 80, 81, 84, 98–101] and either concurrently or independently potentiating activation of FOXO1α [77, 84], a key transcription factor for the induction of the gluconeogenic enzymes, G6Pase and PEPCK [93, 102] (and for the induction of enzymes that damage respiratory complexes leading to increased ROS production and subsequent insulin resistance) [103]. Increases in JNK transcription can be induced by incomplete FFA oxidation and ROS production, as described above, as well as by local or circulating cytokines [53, 99] and JNK is a strong stimulus for activation of FOXO1α [84]. Liver FOXO1α activation has been coupled to the insulin resistant diabetic phenotype [58–64, 87, 104]. Moreover, hepatic PGC1α, a transcription factor that associates with FOXO1α to induce transcription of gluconeogenic enzymes, induces hepatic insulin resistance and is itself elevated in diabetes [89–91, 93–96, 105]. Increased PGC1α level is also a strong activator of fatty acid oxidation in liver and this increased activity can function to maintain the proinflammatory/gluconeogenic state by providing for excess fat oxidation as described above. Increases in hepatic SOCS3, by JNK or other cytokine/oxidative stress factors, contribute to increased hepatic glucose output by inducing insulin resistance (by inactivating IRS1/2 and/or inducing PGC1α and FOXO1) [106]. Low-level activation of NFκB stimulates the production of IL-1β, IL-6, and TNFα that can in turn a) stimulate the activation and/or synthesis of NFκB, JNK, and SOCS3 and b) additionally directly inhibit the insulin signaling pathway [53, 80, 99, 101]. Therefore, simultaneous increases in NFκB, JNK, and SOCS3 can contribute to a strong and potentially self-sustaining pro-gluconeogenic/pro FFA oxidative environment.

Activation of NFκB, JNK, and SOCS3 also stimulates hepatic lipid production. Increases in hepatic JNK (via IL-6, TNFα, FFA, or oxidative stress) or NFκB can induce SOCS3 transcription that in turn can induce SREBP1, a potent transcription factor for several lipogenic enzymes within the liver [57]. Furthermore, this activity may be enhanced by PGC1β, a strong coactivator of SREBP1 to stimulate lipogenic gene expression [89–91, 93–96, 107]. Hepatic reduction of JNK or NFκB [108–110] or suppression of SOCS3 [101] reduces fatty liver. Additionally, other studies have shown that increases in hepatic ROS level are also a stimulus for SREBP1 synthesis without effect on insulin signaling through IRS-1 or AKT [111–114]. Hepatic insulin resistance is also coupled to enhanced liver PPARγ levels that function to increase lipid synthesis [115–117]. It should be appreciated that hepatic gene knock-out studies, as opposed to suppression studies, for any one of these three proteins may yield results indicating exacerbation instead of reduction of the hepatic gluconeogenic/lipogenic phenotype due to overcompensation of redundant pro-inflammatory pathways [118], however the overwhelming composite of available evidence indicates that moderate reductions of elevated levels of these proteins towards the normal range is coupled to a reduction of the gluconeogenic/lipogenic liver phenotype as outlined above. Finally, another master regulator of hepatic lipogenesis, mTORC1, not only induces lipogenic activity but can potentiate insulin resistance via stimulation of SOCS3 expression [119], which can also stimulate lipogenesis via SREBP1 as described above. Therefore, moderate and simultaneous increases in hepatic ROS [111–114] and/or NFκB, JNK, and SOCS3 can interact to facilitate the induction and maintenance of a more gluconeogenic/FFA oxidative and lipogenic liver via simultaneous induction of the master activators of lipogenesis (PGC1β, SREBP1, and mTORC) and gluconeogenesis (FOXO1, PGC1α), concurrently with induction of FFA-oxidative activity (PGC1α, PPARα), activity of which in turn sustains the increase in ROS and pro-inflammatory protein environment and a vicious cycle is born. These ROS and proinflammatory proteins may be induced by a high fat diet (with consequent over-supply of FFAs to liver) [50–55, 57] or by an altered neuroendocrine organization (e.g., low SCN dopamine and high VMH NE and 5HT activities) independent of a high fat diet that re-programs metabolism favoring a liver biochemistry inducing their production as in the SHR rats held on a regular (low-fat) diet in this study [1]. This neuroendocrine driven hepatic pro-inflammatory state may occur with even moderately increased antecedent incomplete FFA oxidation, mitochondrial dysfunction, ER stress and ROS over-generation.

Although typically viewed as pathology, such an endogenous programmed mechanism for increased hepatic lipid and glucose production can have substantive survival benefits for animals in the wild during seasons of low food availability. During long seasons of low food (and glucose) supply, a programmed induction of increased hepatic lipogenesis and secretion, coincident with increased hepatic glucose output, under the setting of insulin resistance would allow for increased peripheral utilization of mobilized fat and CNS utilization of (hepatic) glucose (that has a predominant requirement for glucose as a fuel source) and such alterations in metabolism would increase odds of organismal survival under this environmental stress. Our previous work indicates that the seasonal obese, insulin resistant state, characterized by increased hepatic lipogenesis and glucose output, can be manifested by alterations in hypothalamic activities characterized by low dopaminergic tone at the area of the SCN and increased noradrenergic and serotonergic tone at the VMH without any alteration in diet [3, 4, 11, 12, 14, 15] and timed bromocriptine treatment reverses both this hypothalamic activity and the heightened hepatic lipogenic/gluconeogenic metabolic state [5, 6, 8–10, 14].

In the present study, bromocriptine decreased abnormally elevated VMH NE and serotonin levels that are known not only to be associated with insulin resistance but actually to induce hyperinsulinemia, insulin resistance, glucose intolerance and fatty liver, increased noradrenergic drive to adipose leading to increased lipolysis (and FFA flux to liver), and increased plasma norepinephrine level potentiating increased gluconeogenesis [11, 12, 14, 15, 120]. Bromocriptine-induced decrease in liver lipid content resulting from decreased plasma insulin levels [5, 6, 10] and present study, decreased hepatic lipogenesis [6, 9] and/or decreased plasma FFA availability [5] may reduce over-stimulated fatty acid oxidation and subsequent incomplete fatty acid oxidation metabolites (acid soluble metabolites) and by-products (such as ceramides, acyl-CoAs, diacylglycerol, and lipid peroxides) [50–55, 57, 78, 79] and subsequent mitochondrial derived ROS [78, 79] collectively thereby simultaneously reducing NFκB, JNK, and SOCS3 levels as observed in this study. Under conditions of insulin resistance, ablation or elimination of any one of these 3 pro-inflammatory protein pathways can be compensated for by up-regulation of the others with maintenance of insulin resistance [98, 101]. However this does not appear to be the case with bromocriptine treatment in this study. Bromocriptine reduced all three pro-inflammatory protein levels concurrently that resultantly can act to contribute to the observed bromocriptine-induced simultaneous reductions in the master activators of lipogenesis and gluconeogenesis as described above. The coupled effects of bromocriptine to concomitantly reduce liver activated FOXO1 (induced increase in FOXO1ser256), G6Pase, and PEPCK levels on the one hand and SREBP1, mTORC, PPARγ, and PGC1β levels on the other offer mechanisms by which bromocriptine treatment reduced both plasma glucose level and hepatic fat content, respectively. The effect of bromocriptine to reduce hepatic gluconeogenic capacity itself may derive in part from its induced reduction of hepatic FFA oxidative capacity as assessed by the reduced levels of hepatic PGC1α and PPARα in bromocriptine-treated animals, levels of which are elevated in insulin resistant states [50–55, 57, 89–91, 93–96]. In addition to its effects to reduce hepatic PGC1α and PPARα, bromocriptine-induced decreases in plasma FFA and liver lipid synthesis may function to reduce liver FFA oxidative capacity and subsequently to reduce ROS production and inflammatory protein(s) synthesis in turn reducing insulin resistance and gluconeogenesis while also breaking the vicious positive feedback cycle on this pathway induced by the ROS.

Since bromocriptine can reverse insulin resistance, glucose intolerance and fattening by its i.c.v. injection, the present observed effects on hepatic glucose and lipid metabolism may be the indirect result of its treatment effects on the entire neuroendocrine axis, not only by influencing the VMH and SCN as described above and reducing neuropeptide Y and corticotropin levels in the paraventricular hypothalamus as previously described [45], and such is the topic of our ongoing investigation. Such centrally elicited effects of bromocriptine do not preclude any additional direct peripheral effects of the therapy to produce the results described herein and such considerations are the focus of our ongoing investigation. The bromocriptine effect on liver insulin signaling pathway proteins and redox status as it relates to its impact on lipogenesis and gluconeogenesis remain as yet undetermined and the results of this study suggest that such investigations are warranted. It should be appreciated that while the small reduction in food consumption with bromocriptine treatment observed in this study may contribute to its overall effects on metabolic status, such small reductions (in low fat content food) are not large enough to explain the large reductions in body fat, liver fat, insulin resistance, or hypertension, and indeed previous studies have demonstrated improvements in insulin resistance and body fat of bromocriptine treated animals without any change in feeding [5, 6, 10].

Respecting neuroendocrine impact on blood pressure in MS, reductions in hyperleptinemia and elevated plasma NE can interact to reduce vasoconstriction and hypertension (reviewed in [1]) and marked improvements in these plasma parameters and of hypertension were observed following bromocriptine treatment of SHR rats. Moreover, we have observed a strong hypertensive effect of VMH NE plus 5HT infusion into the VMH of normal rats [10] and in the present study bromocriptine treatment reduced elevated VMH NE and 5HT activities in these SHR rats to normal levels. Previous studies have likewise documented an anti-hypertensive effect of bromocriptine in SHR rats and investigations into mechanisms of this bromocriptine-induced effect suggest both complex central and peripheral actions of the agent to reduce sympathetic tone, plasma NE level, and vasoconstriction [106, 121–127].

In summary, timed bromocriptine treatment reduces overactive noradrenergic and serotonergic activities at the VMH of hypertensive, obese, insulin resistant SHR rats, activities of which have previously been demonstrated to predispose to insulin resistance, hypertension, and fattening ([7], reviewed in [1]). This bromocriptine treatment also reduces hypertension and neuroendocrine stimuli for hepatic insulin resistance and lipid accumulation, particularly increased sympathetic drive, hyperinsulinemia, hyperleptinemia, and reduced plasma adiponectin. Its effects to reduce liver lipid content and gluconeogenic capacity at the level of the liver are coupled to a reduction of multiple activated pro-inflammatory protein pathways that concurrently induce transcriptional master activators of lipogenesis and gluconeogenesis. Simultaneously, such bromocriptine treatment also reduces major transcription factors that increase FFA oxidative capacity of the liver, which in and of itself may contribute to the drug-induced reduction in gluconeogenic enzyme levels. Available evidence suggests that these peripheral effects of bromocriptine are likely functionally linked to its impact on hypothalamic, particularly SCN and VMH activities.

## Authors’ original submitted files for images

Below are the links to the authors’ original submitted files for images. Authors’ original file for figure 1 Authors’ original file for figure 2 Authors’ original file for figure 3 Authors’ original file for figure 4 Authors’ original file for figure 5 Authors’ original file for figure 6 Authors’ original file for figure 7 Authors’ original file for figure 8 Authors’ original file for figure 9

## Abbreviations

5HIAA: 5-hydroxy-indoleacetic acid
5HT: 5-hydroxytryptamine
ANOVA: Analysis of variance
AUC: Area under curve
CNS: Central nervous system
CRH: Corticotropin-releasing hormone
ECL: Enhanced chemiluminescence
ER: Endoplasmic reticulum
FFA: Free fatty acids
GSIS: Glucose stimulated insulin secretion
HALO: Hours after light onset
HOMA-IR: Homeostatic model assessment – insulin resistance
HVA: Homovanillic acid
Icv: Intracerebroventricular
i.p.: Intraperitoneal
MS: Metabolic syndrome
MHPG: 3-methoxy-4-hydroxy-phenylglycol
NE: Norepinephrine
NPY: Neuropeptide Y
PVDF: Polyvinylidene difluoride
PVN: Paraventricular nucleus
ROS: Reactive oxygen species
S: Serotonin
SEM: Standard error of the mean
SCN: Suprachiasmatic nuclei
SHR: Spontaneously hypertensive rat
VMH: Ventromedial hypothalamus.
