# Erratum: Neuroendocrine and metabolic components of dopamine agonist amelioration of metabolic syndrome in SHR rats

**DOI:** 10.1186/s13098-015-0056-x

**Published:** 2015-07-04

**Authors:** Michael Ezrokhi, Shuqin Luo, Yelena Trubitsyna, Anthony H. Cincotta

**Affiliations:** VeroScience LLC, Tiverton, RI 02878 USA

## Erratum

After publication of this manuscript [1], we noted errors to the labels of Fig. 7. The Y-axis of panel C was incorrectly labelled as “Plasma Adiponectin, ng/ml” instead of “PEPCK, % of Vehicle”. (Please see a corrected version of Fig. 7 below).

**Fig. 7:**
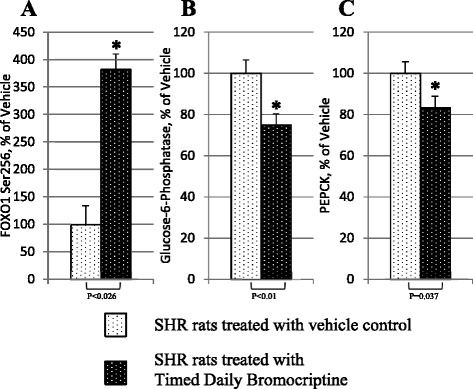
Impact of timed daily bromocriptine or vehicle administration on gluconeogenic pathway regulators – FOXO1 phosphorylated at Ser256 (Panel **a**), glucose-6-phosphatase (Panel **b**), and PEPCK (Panel **c**). Proteins we quantified by Western blotting. Values are means ± SEM of 8 animals in each group. *Difference is statistically significant; P values are noted under each panel
