# Supplementary material for: Neuroendocrine and metabolic components of dopamine agonist amelioration of metabolic syndrome in SHR rats
Source: Diabetol Metab Syndr. 2014 Sep 25;6:104. doi: 10.1186/1758-5996-6-104 (PMC4416398; doi:10.1186/1758-5996-6-104)
Supplement: Supplementary file 1 — Authors’ original file for figure 1 [file 13098_2014_418_MOESM1_ESM.pdf]

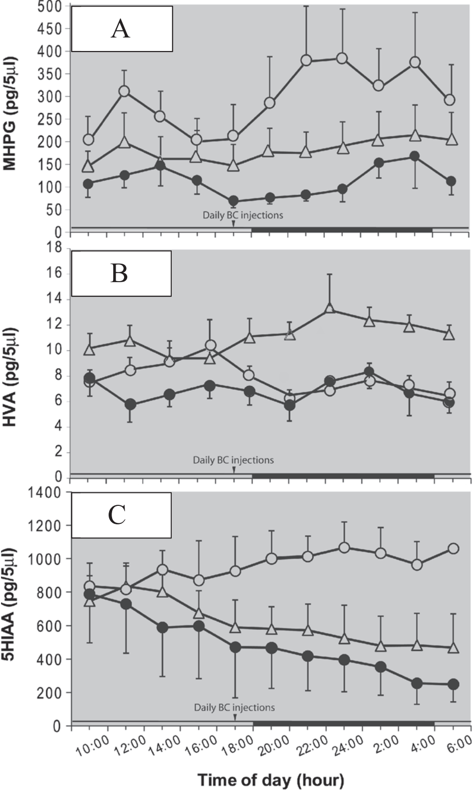

○ SHR rats treated with vehicle control
● SHR rats treated with Timed Daily Bromocriptine
△ Wistar rats
