# Supplementary material for: Neuroendocrine and metabolic components of dopamine agonist amelioration of metabolic syndrome in SHR rats
Source: Diabetol Metab Syndr. 2014 Sep 25;6:104. doi: 10.1186/1758-5996-6-104 (PMC4416398; doi:10.1186/1758-5996-6-104)
Supplement: Supplementary file 2 — Authors’ original file for figure 2 [file 13098_2014_418_MOESM2_ESM.pdf]

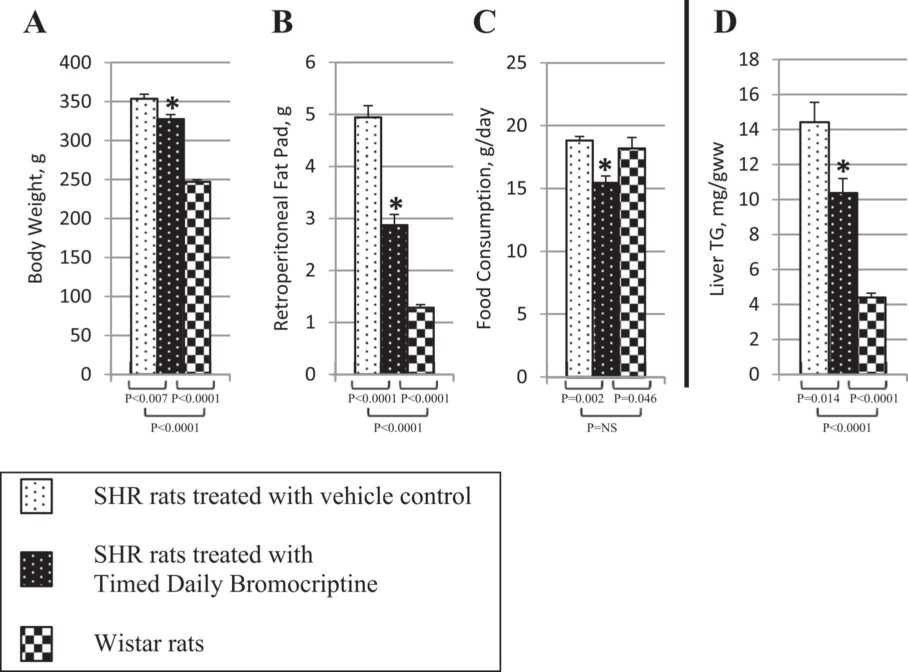

A
B
C
D
Body Weight, g
Retroperitoneal Fat Pad, g
Food Consumption, g/day
Liver TG, mg/gww
P<0.007
P<0.0001
P<0.0001
P<0.0001
P<0.0001
P<0.0001
P=0.002
P=0.046
P=NS
P=0.014
P<0.0001
P<0.0001
SHR rats treated with vehicle control
SHR rats treated with Timed Daily Bromocriptine
Wistar rats
