# Supplementary material for: Neuroendocrine and metabolic components of dopamine agonist amelioration of metabolic syndrome in SHR rats
Source: Diabetol Metab Syndr. 2014 Sep 25;6:104. doi: 10.1186/1758-5996-6-104 (PMC4416398; doi:10.1186/1758-5996-6-104)
Supplement: Supplementary file 3 — Authors’ original file for figure 3 [file 13098_2014_418_MOESM3_ESM.pdf]

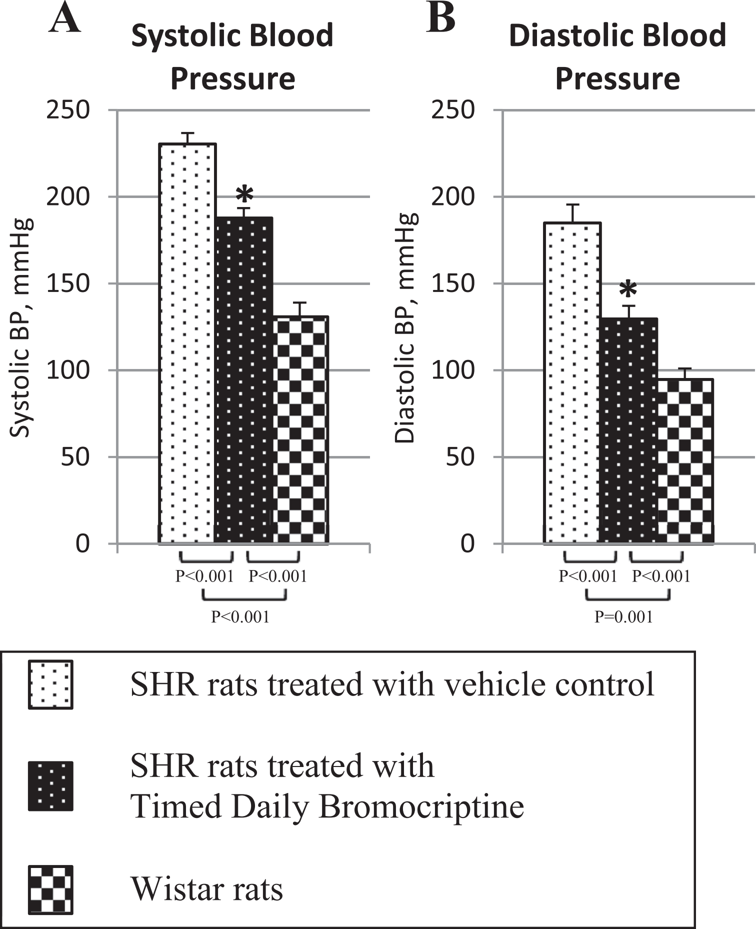
A
Systolic Blood Pressure
250
200
150
100
50
0
Systolic BP, mmHg
*
P<0.001
P<0.001
P<0.001
B
Diastolic Blood Pressure
250
200
150
100
50
0
Diastolic BP, mmHg
*
P<0.001
P<0.001
P=0.001
SHR rats treated with vehicle control
SHR rats treated with Timed Daily Bromocriptine
Wistar rats
