# Supplementary material for: Neuroendocrine and metabolic components of dopamine agonist amelioration of metabolic syndrome in SHR rats
Source: Diabetol Metab Syndr. 2014 Sep 25;6:104. doi: 10.1186/1758-5996-6-104 (PMC4416398; doi:10.1186/1758-5996-6-104)
Supplement: Supplementary file 4 — Authors’ original file for figure 4 [file 13098_2014_418_MOESM4_ESM.pdf]

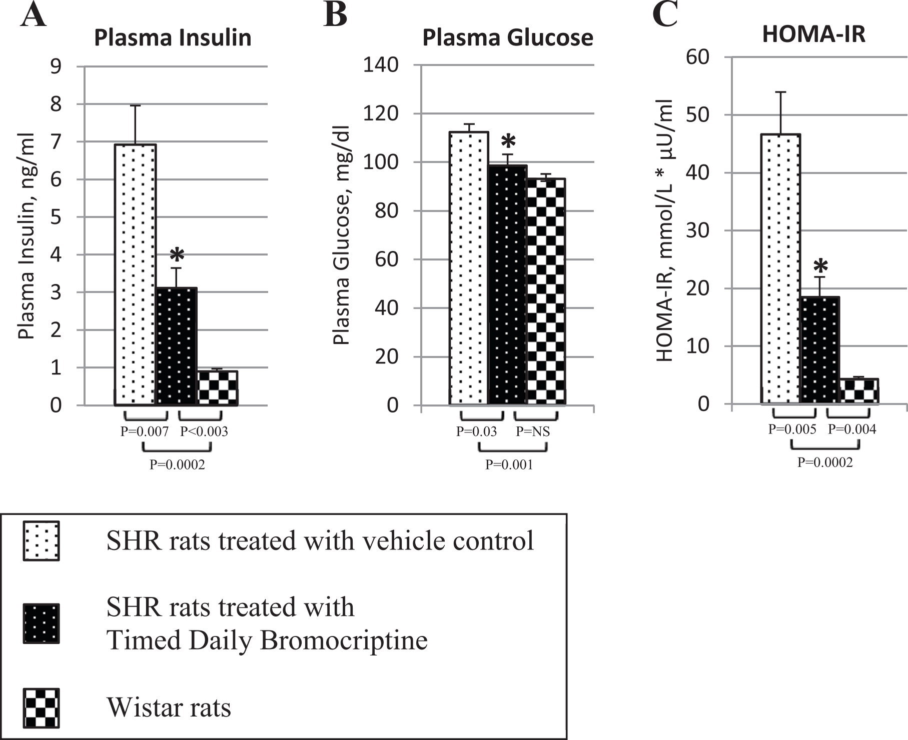

A
Plasma Insulin
Plasma Insulin, ng/ml
0
1
2
3
4
5
6
7
8
9
*
P=0.007
P<0.003
P=0.0002
B
Plasma Glucose
Plasma Glucose, mg/dl
0
20
40
60
80
100
120
140
*
P=0.03
P=NS
P=0.001
C
HOMA-IR
HOMA-IR, mmol/L * µU/ml
0
10
20
30
40
50
60
*
P=0.005
P=0.004
P=0.0002
SHR rats treated with vehicle control
SHR rats treated with Timed Daily Bromocriptine
Wistar rats
