# Supplementary figures and images for: Neuroendocrine and metabolic components of dopamine agonist amelioration of metabolic syndrome in SHR rats
Source: Diabetol Metab Syndr. 2014 Sep 25;6:104. doi: 10.1186/1758-5996-6-104 (PMC4416398; doi:10.1186/1758-5996-6-104)

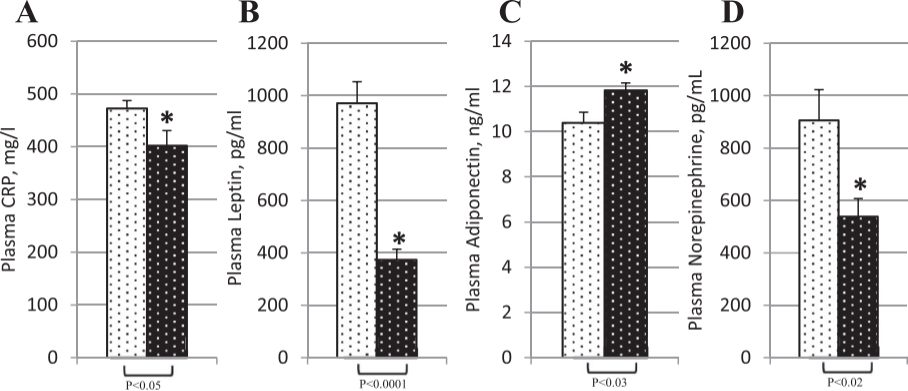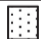

SHR rats treated with vehicle control

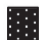

SHR rats treated with  
Timed Daily Bromocriptine

Supplement: Supplementary file 5 — Authors’ original file for figure 5 [file 13098_2014_418_MOESM5_ESM.pdf]

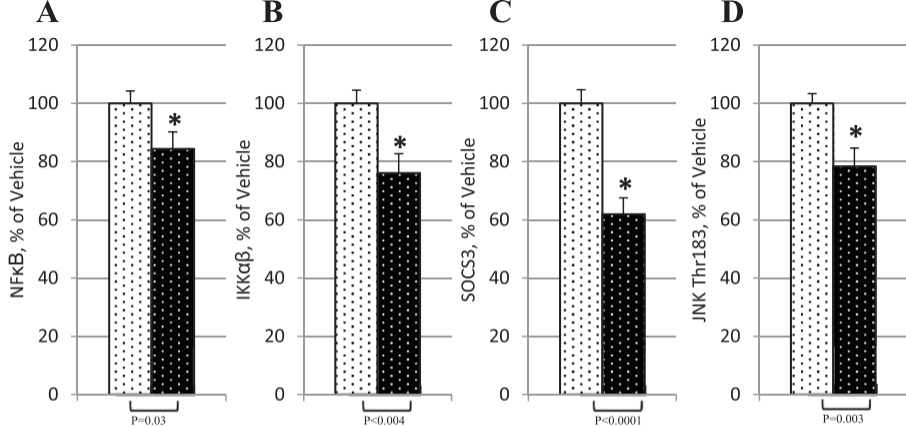

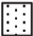 SHR rats treated with vehicle control

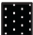 SHR rats treated with  
Timed Daily Bromocriptine

Supplement: Supplementary file 6 — Authors’ original file for figure 6 [file 13098_2014_418_MOESM6_ESM.pdf]

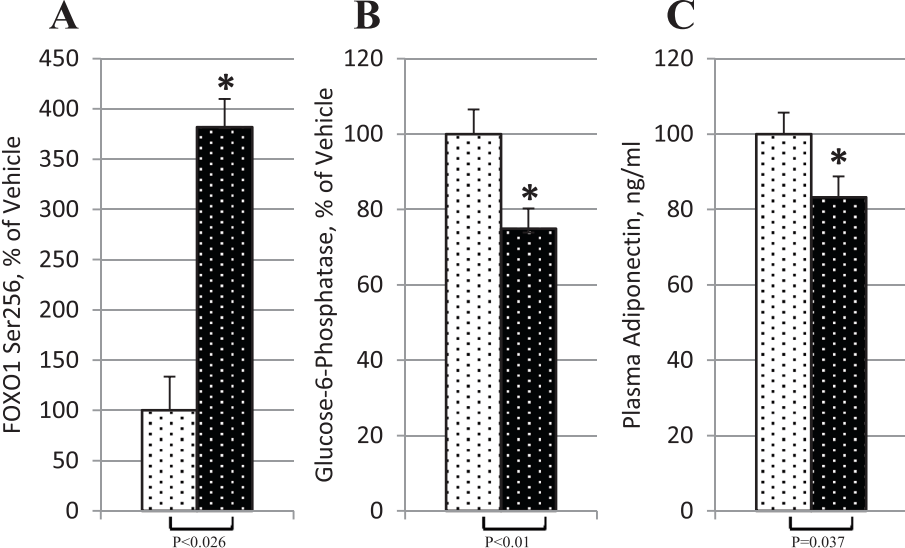

Supplement: Supplementary file 7 — Authors’ original file for figure 7 [file 13098_2014_418_MOESM7_ESM.pdf]

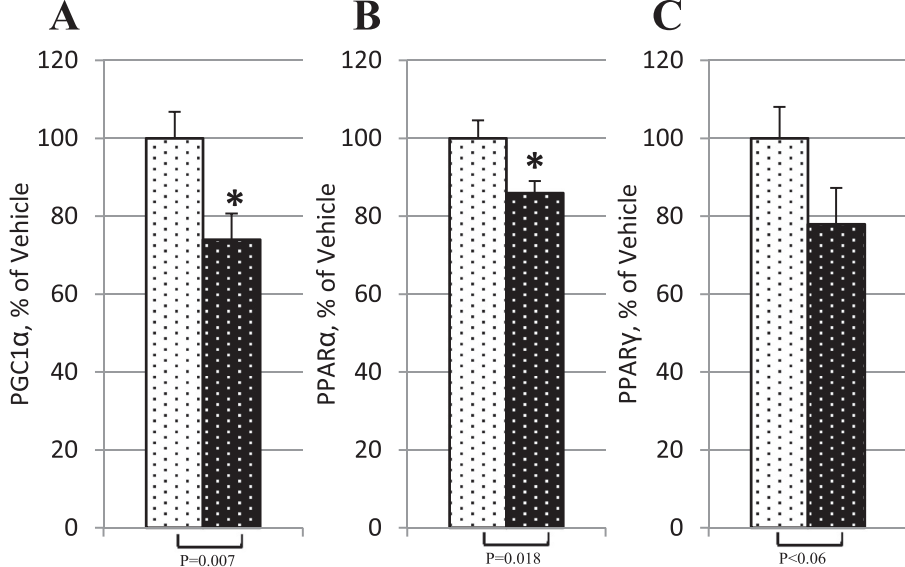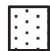

SHR rats treated with vehicle control

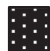

SHR rats treated with  
Timed Daily Bromocriptine

Supplement: Supplementary file 8 — Authors’ original file for figure 8 [file 13098_2014_418_MOESM8_ESM.pdf]

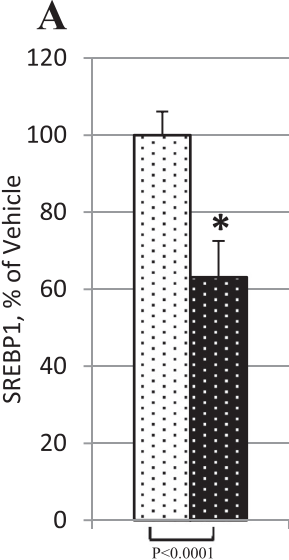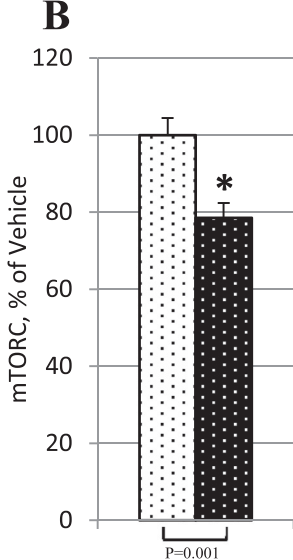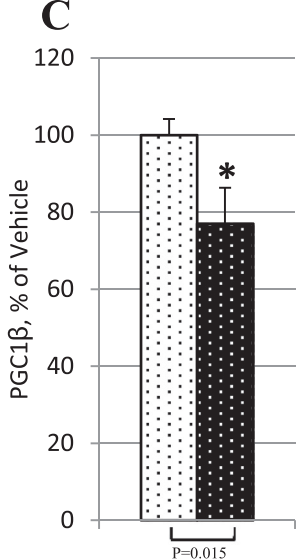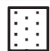

SHR rats treated with vehicle control

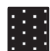

SHR rats treated with  
Timed Daily Bromocriptine

Supplement: Supplementary file 9 — Authors’ original file for figure 9 [file 13098_2014_418_MOESM9_ESM.pdf]
